# Triglycerides/Glucose Index Is Associated with Sperm Parameters and Sperm DNA Fragmentation in Primary Infertile Men: A Cross-Sectional Study

**DOI:** 10.3390/metabo12020143

**Published:** 2022-02-03

**Authors:** Federico Belladelli, Luca Boeri, Edoardo Pozzi, Giuseppe Fallara, Christian Corsini, Luigi Candela, Walter Cazzaniga, Daniele Cignoli, Luca Pagliardini, Alessia D’Arma, Paolo Capogrosso, Eugenio Ventimiglia, Francesco Montorsi, Andrea Salonia

**Affiliations:** 1 Division of Experimental Oncology, Unit of Urology, URI, IRCCS Ospedale San Raffaele, 20132 Milan, Italy; luca.boeri@hotmail.it (L.B.); pozzi.edoardo@hsr.it (E.P.); fallara.giuseppe@hsr.it (G.F.); corsini.christian@hsr.it (C.C.); candela.luigi@hsr.it (L.C.); cazzanigaw@gmail.com (W.C.); cignoli.daniele@hsr.it (D.C.); darma.alessia@hsr.it (A.D.); ventimiglia.eugenio@hsr.it (E.V.); montorsi.francesco@hsr.it (F.M.); 2 Department of Urology, University Vita-Salute San Raffaele, 20132 Milan, Italy; 3 Department of Urology, Foundation IRCCS Ca’ Granda—Ospedale Maggiore Policlinico, University of Milan, 20122 Milan, Italy; 4 Division of Genetics and Cell Biology, Reproductive Sciences Laboratory, IRCCS Ospedale San Raffaele, 20132 Milan, Italy; Pagliardini.luca@hsr.it; 5 Department of Urology and Andrology, Ospedale di Circolo and Macchi Foundation, 21100 Varese, Italy; paolo.capogrosso@gmail.com

**Keywords:** infertility, semen parameters, insulin resistance, triglycerides/glucose index, risk factors

## Abstract

Study question: we aimed to investigate the relationship between the tyg index and both semen and hormonal characteristics in a cohort of primary infertile men. Summary answer: almost one in two primary infertile men presented with a triglycerides/glucose index (tyg) suggestive of insulin resistance (ir). overall, patients with tyg suggestive of ir showed worse clinical, hormonal, and semen parameters. What is already known: male factor infertility (MFI) is often associated with metabolic disorders such as diabetes mellitus and metabolic syndrome, where insulin resistance (IR) plays a relevant pathological role. Recently, TyG has been suggested as a user-friendly IR marker. Study Design: serum hormones and the sperm DNA fragmentation index (SDF) were measured in every patient. The semen analysis was based on 2010 WHO reference criteria. Glucose and insulin levels were measured for every man after a 12-h overnight fast, and the homeostatic model assessment index (HOMA-IR) was then calculated and categorized using a 2.6 threshold. Similarly, fasting glucose and triglycerides levels were measured and the TyG index was calculated and categorized using an 8.1 threshold. Descriptive statistics and logistic regression models tested the association between the TyG and semen and hormonal characteristics. Participants: complete demographic, clinical, and laboratory data from 726 consecutive white European primary infertile men were considered for this analysis. Main results and the role of chance: the median (IQR) age was 39 (35–43) years. A TyG and HOMA suggestive for IR was found in 339 (46.6%) and 154 (21.2%) men, respectively. During the Spearman’s test, the TyG index was highly correlated with HOMA-IR (rho = 0.46, *p* < 0.001). Compared to men with a normal TyG, men with TyG > 8.1 were older, had greater BMI and CCI scores, and lower total testosterone and sperm concentration, but higher DFI, and presented a greater proportion of NOA (all *p* < 0.01). The multivariable logistic regression analysis showed that men with TyG > 8.1 were at higher risk of SDF > 30 (OR 1.92 (CI: 1.2–2.9)) and NOA (OR 1.78 (CI: 1.1–2.8)). Wider implications of the findings: the Tyng index may act as a reliable marker of IR in the clinical work-up of primary infertile men in real-life settings.

## 1. Introduction

It is estimated that approximately 15% of couples are unable to achieve a spontaneous pregnancy after 1 year of regular, unprotected sexual intercourse and are classified as infertile. Among these, a male factor cause of infertility (MFI) can be recognized in approximately 50% of cases [1,2]. In this context, it is of critical importance that every infertile man may receive a comprehensive evaluation by specialists in male reproduction to identify potentially treatable causes of MFI [1,3]. Several conditions have been associated with MFI, and tough infertility is still idiopathic in nature in about 30–40% of men [1]. Among the treatable causes of male infertility, semen infections, smoking habits, alcohol consumption, abnormal DNA methylation, bacterial and viral infections, and recreational substance use have been consistently associated with impaired sperm quality [4,5,6,7]. Furthermore, metabolic disorders such as prediabetes, diabetes mellitus (DM), and metabolic syndrome (MetS) are commonly found in infertile men [8,9,10]. In this context, DM and insulin resistance (IR) have been observed to negatively affect sperm quality and hormonal values, thus contributing to MFI [11]. The underlying mechanisms of this association include the impaired function of the hypothalamic–pituitary–gonadal axis, increased DNA damage, oxidative stress, and disrupted sympathetic innervation [12]. The alteration of the energy balance through the elevation in the levels of inflammatory cytokines and oxidative stress can affect male reproductive functions [13]. Furthermore, IR, along with MFI, has been associated with an increased risk of cardiovascular diseases (CVDs) [11,14]. Therefore, the precise evaluation of IR appears to be of clinical relevance in the diagnostic work-up of infertile men.

The glucose clamp technique is currently considered the gold standard test for detecting IR [15,16], but alternative methods based on insulin and glucose levels have been developed, such as the homeostatic model assessment (HOMA-IR) and the quantitative insulin sensitivity check index (QUICKI) [17]. However, the insulin test is expensive and not routinely performed worldwide [18,19]. A simple, inexpensive and easily accessible marker of IR has also been introduced into clinical use, i.e., the triglycerides/glucose index (TyG), which is calculated by fasting serum glucose and triglyceride values [20]. Previous studies have shown that the TyG index may be considered a good surrogate marker of IR as compared with HOMA-IR in different clinical settings, thus including men with erectile dysfunction [21,22,23].

Following the critical association observed between IR and impaired reproductive function, along with the shared higher risk of future CVDs among infertile men and individuals with data suggestive for IR, the identification of cheap, easily accessible markers of IR that can be included in the management work-up of infertile men—both in the diagnostic phase and the scheduling of personalized preventive strategies—is of primary clinical importance.

Hence, we aimed to investigate the association between the TyG index with semen parameters and the hormonal milieu in a relatively large and homogenous cohort of non-Finnish, white European men seeking medical help for primary couple’s infertility at a single tertiary referral center.

## 2. Results

Table 1 depicts the clinical characteristics of the entire cohort of patients and segregated them according to the TyG index score. Of 726 patients, 339 (46.7%) men had a TyG index ≥ 8.1. Infertile men with a TyG index ≥ 8.1 were older (37 vs. 39 years), had a higher BMI (24.5 vs. 25.6 kg/m^2^), a higher rate of CCI ≥ 1 (13 vs. 45), and a longer duration of infertility compared to those with a normal TyG index (20 vs. 24 months) (all *p* < 0.001) (Table 1).

Moreover, participants with a TyG index ≥ 8.1 showed higher total cholesterol (182 vs. 203 mg/dL), insulin levels (6.6 vs. 9.0 mUI/L), and HOMA-IR index (1.4 vs. 2) than those with a TyG index < 8.1 (all *p* < 0.001) (Table 2). Conversely, tT was lower in men with a TyG index suggestive of IR than those with a normal TyG index (4.5 vs. 4.2 ng/mL), with a greater prevalence of patients with tT suggestive of hypogonadism (55 vs. 78) (all *p* ≤ 0.01).

Overall, 95 (28.0%) patients had both indexes suggestive of IR (Table 2).

Table 3 reports the sperm parameters of the whole cohort and segregated them according to TyG index scores. Patients with a TyG index ≥ 8.1 depicted a lower sperm concentration, along with a higher rate of both oligozoospermia and iNOA (all *p* ≤ 0.01). Moreover, SDF was higher and more frequently pathologic in men with a TyG index ≥ 8.1 (all *p* ≤ 0.01) (Table 3).

Using the Spearman’s correlation, it was found that TyG was highly correlated with the HOMA-IR score (rho = 0.46, *p* < 0.001).

Table 4 reports univariable and multivariable logistic regression analysis testing the association between the predictors and oligozoospermia, SDF > 30%, and iNOA.

The multivariable logistic regression analysis revealed that men with a TyG index ≥ 8.1 (OR 1.58; *p* = 0.03), smaller TV (OR 0.89; *p* < 0.01) and higher FSH values (OR 1.21; *p* < 0.001) were at higher risk of oligozoospermia, after accounting for age, BMI and CCI.

Similarly, a TyG index ≥ 8.1 (OR 1.78; *p* < 0.01), smaller TV (OR 0.91; *p* < 0.001), higher FSH values (OR 1.11; *p* < 0.01) and higher CCI scores (OR 2.05; *p* = 0.03) were independently associated with iNOA, after adjusting for age and BMI.

Older age (OR 1.1; *p* = 0.01) and smaller TV (OR 0.93; *p* < 0.01) were associated with SDF. Similarly, infertile men with a TyG index suggestive of IR had a 2-fold higher risk of pathological SDF (*p* < 0.01) compared to those with a normal TyG index, after accounting for BMI, CCI and FSH values (Table 4).

## 3. Discussion

A growing number of clinical data over the last decade showed that primary infertile men are less healthy than their fertile counterparts, thus outlining the importance of planning a more comprehensive and tailored management for these fragile individuals [24,25,26]. In this context, finding effective biomarkers which may help and personalizing preventive strategies for those infertile men who are at an actual higher risk of developing chronic diseases or cancers emerged to be clinically relevant. Among other potential diseases, prediabetes, hypertension and MetS have been observed to occur more frequently in infertile men, and certainly at an earlier phase in life, with all possible subsequent long-term sequelae [11,15,27].

Here, we applied a new marker of IR, termed the TyG index, which emerged as a simple, inexpensive and user-friendly biomarker, obtained by calculating fasting serum glucose and triglyceride values [20]. Of clinical relevance, we observed that 47% of infertile men in our large and homogenous cohort had TyG index values suggestive of IR. Infertile men with a TyG index ≥ 8.1 showed worse clinical, hormonal and sperm parameters than those with a TyG index not suggestive of IR. Similarly, the TyG index was independently associated with oligozoospermia, iNOA and higher SDF levels, after accounting for common clinical and hormonal variables.

Several studies have investigated the impact of IR in infertile men. This study was motivated by a large amount of literature showing the detrimental impact of IR on the clinical and sperm parameters of infertile men and by the urgent need of identifying an easy and accessible marker of IR to be used in everyday clinical practice. Ventimiglia et al., for instance, analyzed data from 1337 primary infertile men and found MetS in 128 (9.6%) participants. Infertile men with MetS were older, had a greater CCI score and lower levels of tT compared to men without MetS [9]. Lotti et al. investigated the metabolic profile of 351 infertile men, of which 7.7% were diagnosed with MetS. Patients with IR had a lower rate of normal semen morphology during multivariable analysis, accounting for age and hormonal profile [28].

Obesity and DM, both clinical conditions associated with IR, are known for their negative impact toward male reproductive function. Excessive peripheral adipose tissue results in increased tT to estradiol aromatization, which eventually leads to secondary hypogonadism and reproductive axis derangement. Increased levels of pro-inflammatory molecules such as TNF-α, IL-6 and C-reactive protein have been found in obese patients, a further condition associated with IR; systemic inflammation associated with those factors may lead to an increased level of reactive oxygen species that can negatively impact semen quality [13,14,29]. Similarly, it has been shown that the sperm plasma membrane and acrosome are affected by serum insulin levels [30]. Therefore, in the case of IR or insulin deficiency, spermatogenesis is altered and biopsies of diabetic patients revealed testicular impairments [31,32]. Agbaje et al. compared semen samples of insulin-dependent diabetic patients with healthy controls and found a significantly lower semen volume and higher sperm DNA fragmentation rates among diabetic patients [33]. Similarly, in a subsequent study with 500 male partners, progressive motility and morphology were found to be significantly impaired in diabetic men compared to controls [34]. La Vignera et al. matched 32 patients with type 1 DM with 20 healthy controls; they observed that a lower sperm concentration and progressive motility were found among young diabetic men [35]. Different mechanisms are considered to contribute to the negative impact of IR on male reproductive function, among which increased ROS and oxidative stress are prominent once again. Increased oxidative stress is supposed to affect several cellular signaling pathways, for instance, apoptosis, autophagy, mitophagy, endoplasmic reticulum stress, inflammation, and angiogenesis that ultimately lead to infertility in diabetic men [32,36].

As said, from a clinical standpoint, it should be recognized that men with parameters suggestive for IR, both from the general and infertile population, are at higher risk of developing future cardiovascular and metabolic disorders later in life [14]. Therefore, the accurate investigation of IR in the specific cohort of infertile men appears of utmost importance not only for investigating a potential treatable cause of MFI in terms of sperm and hormonal parameters but also for adopting preventive strategies to avoid potential future diseases in terms of general well-being. HOMA-IR is currently considered the gold standard for IR investigation in clinical practice. Calderòn et al. investigated twenty severely obese men before and 2 years after bariatric surgery. The authors found that sperm characteristics did not improve after bariatric surgery despite the beneficial changes in reproductive hormones and HOMA values [37]. Moreover, previous authors have observed that fatty acids concentration and HOMA-IR levels were higher in diabetic patients with vasculogenic erectile dysfunction compared to controls [38]. A major limitation of HOMA-IR is that insulin testing is a hard-to-reach and scarce test, especially in low-income populations, and is limited in terms of reproducibility [16]. In this context, the TyG index, based on fasting glucose and triglyceride values, which are low cost and easily accessible biochemistry tests [39,40], has been introduced as an alternative marker of IR. Several studies have shown a positive correlation between HOMA-IR and the TyG index [22] in clinical practice and, of note, some reports have shown that the TyG index is an even better diagnostic tool for IR than the HOMA score in selected patients [16,41,42]. Recently, Yilmaz et al. evaluated the role of the TyG index in a cohort of 152 males with sexual dysfunction [23]; the authors found that men with erectile dysfunction had a higher TyG index and HOMA scores compared to men without ED. Moreover, they showed that a TyG index > 4.78 independently predicted ED.

Our study is novel since it is the first to explore the association between the TyG index and semen and hormonal parameters in a cohort of infertile men. We confirm the known negative effect of IR on reproductive function, since infertile men with a TyG index ≥ 8.1 had worse clinical, hormonal and sperm parameters than those with a TyG index not suggestive for IR. Moreover, the TyG index was independently associated with oligozoospermia, iNOA and SDF, after accounting for common clinical and hormonal variables, thus outlining the relevance of IR testing in infertile men. Conversely, despite being strongly correlated, we observed that only 28% of our patients had a concomitant pathological TyG index and HOMA scores. This last finding (i.e., almost one out of three infertile males being present with concomitant HOMA-IR and TyG indexes suggestive for IR, despite being phenotypically healthy young men) might suggest that the TyG index may still eventually overestimate the proportion of primary infertile men with IR as compared with HOMA; as a consequence, these results also suggest that further studies are needed to identify the best cutoff of the TyG index, at least to identify infertile men with IR, and all consequent clinical features.

Our study is not devoid of limitations. First, this was a single center-based study, raising the possibility of selection biases, thus it may potentially have an impact on the generalizability of the findings. Second, the analyses were implemented in a cross-sectional setting without a comparison with a same-ethnicity, age-matched cohort of fertile individuals. Third, the TyG index cutoff of IR was based on previous studies [20,21] and we cannot exclude that different values would better categorize infertile men in terms of IR status. To this aim, the comparison with a control group of fertile men is needed.

## 4. Materials and Methods

### 4.1. Study Population

The analyses of this cross-sectional study were based on a cohort of 987 men seeking first medical help at a single academic center for primary couple’s infertility between September 2012 and September 2020. According to the WHO criteria, infertility is defined as not conceiving a pregnancy after at least 12 months of unprotected intercourse regardless of whether or not a pregnancy ultimately occurs [43]. Primary infertility is defined as when a couple has never been able to conceive [43]. Patients were only enrolled if they were ≥18 and ≤55 years old and had pure MFI. MFI was defined after a comprehensive diagnostic evaluation of all the female partners by expert gynaecologists.

### 4.2. Patient Assessment

All men were assessed with a thorough medical history, including age and comorbidities. Health-significant comorbidities were scored with the Charlson comorbidity index (CCI) [44,45], further categorized as CCI = 0 vs. ≥1. The measured body mass index (BMI), defined as weight in kilograms by height in square meters, was obtained for each patient [9]. Testes volume (TV) was assessed in all cases using Prader’s orchidometer estimation; for the specific purpose of this study, we calculated the mean value between the two sides [46]. Varicocele was also clinically assessed in every patient [47].

### 4.3. Blood Analyses

Venous blood samples were drawn from each patient between 7 AM and 11 AM after a 12-h overnight fast [48]. Follicle-stimulating hormone (FSH), luteinizing hormone (LH), total testosterone (tT), inhibin B (InhB), sex hormone-binding globulin (SHBG), prolactin, fasting glucose, total cholesterol, triglycerides, and insulin levels were measured. The HOMA-IR index was calculated based on glucose and insulin levels and subsequently classified using a 2.6 threshold, previously shown to be an accurate predictor of IR [19,49]. Glucose and triglycerides levels were then used to calculate the TyG index. TyG was classified using 8.1 as a threshold, previously shown to be an accurate predictor of IR [20,21]. Hypogonadism was defined for tT < 3 ng/mL [50].

According to our internal diagnostic protocol, chromosomal analysis and genetic testing were performed in every infertile man (i.e., karyotype analysis and Y-chromosome microdeletions and cystic fibrosis mutations tests) [51].

### 4.4. Semen Analyses

All patients underwent two consecutive semen analyses at least 3 months apart; for the specific purposes of this study, we considered semen volume, sperm concentration, progressive sperm motility, and morphology [52]. According to 2010 WHO reference criteria, oligozoospermia is defined as <15 million sperm per ml, asthenozoospermia is defined as <32% progressive motility, and teratozoospermia is defined as <4% of normal forms. Non-obstructive azoospermia (NOA) is defined as the absence of sperm due to non-obstructive causes in two consecutive semen analyses after the centrifugation of the sample [1]. Idiopathic NOA (iNOA) was determined after excluding all known causes of NOA.

The sperm DNA fragmentation (SDF) index, as measured by a sperm chromatin structure assay (SCSA), was assessed in each man. SDF was considered pathological if >30% [53].

The same laboratory was used for the analyses of all parameters in all patients.

We excluded 261 (26.4%) men because they missed one or more of the entry criteria (i.e., abnormal genetic tests (any type) (*n* = 38; 3.8%), had symptoms suggestive of genitourinary infections (*n* = 25; 2.5%) or positive semen or urine cultures (*n* = 151; 15.2%), had a history of assisted reproductive techniques (ART) (any type) during the preceding year (*n* = 7; 0.7%), and if they were already known to have glycemic values above the previously reported threshold or had a previous diagnosis of either DM or were under hypoglycemic medications (*n* = 40; 4.5%)). Similarly, none of the considered patients had a recent history (i.e., over the last 6 months) or were under medication to improve semen parameters. A convenient sample of 726 infertile men was considered for the final statistical analyses.

Data collection followed the principles outlined in the Declaration of Helsinki; all patients signed an informed consent form agreeing to deliver their own anonymous information for future studies. The study was approved by the IRCCS San Raffaele Hospital Ethical Committee (Prot. 2014—Pazienti Ambulatoriali).

### 4.5. Statistical Analysis

The distribution of data was tested with the Shapiro–Wilk test [54]. Data are presented as medians (interquartile range; IQR) or frequencies (proportions). The Mann–Whitney test and the chi-square test were used to compare baseline clinical and demographic characteristics, hormonal values, and semen parameters between those individuals with a normal vs. pathological TyG index [55].

Univariable and multivariable logistic regression analyses tested the association between clinical, hormonal, and metabolic characteristics (i.e., age, BMI, CCI, FSH, TV, and the TyG index) with oligozoospermia, iNOA and SDF > 30% [56].

Statistical analyses were performed using SPSS v.26 (IBM Corp., Armonk, NY, USA). All tests were two sided and the statistical significance level was determined at *p* < 0.05.

## 5. Conclusions

Given the importance of IR in terms of overall men’s health, findings from this cross-sectional study showed that 47% of primary infertile men had TyG index values suggestive for IR. Patients with a TyG index suggestive for IR showed worse clinical, hormonal and semen parameters compared to those with lower values. The TyG index may act as a novel, cheap and easily accessible marker of IR in the clinical work-up in primary infertile men in everyday clinical practice, thus supporting an easier long-term follow-up of patients at higher risk of chronic dysmetabolic comorbidities. However, further studies are needed to define the most reliable cut-off, since only 28% of the whole cohort had concomitant pathological HOMA-IR scores and TyG indexes, thus suggesting that the latter user-friendly and cost-effective parameter may overestimate the actual proportion of primary infertile men with IR.

## Figures and Tables

**Table 1:** Demographic characteristics of the whole cohort of infertile patients according to TyG index suggesting insulin resistance (*N* = 726).

	Overall	TyG Index < 8.1	TyG Index ≥ 8.1	*p* Value *
No. of patients [No. (%)]	726 (100)	387 (53.4)	339 (46.7)	
Age (years)				<0.001
Median (IQR)	38.0 (35–42)	37.0 (34–41)	39.0 (35–43)	
Range	19–55	22–55	19–55	<0.001
BMI (kg/m^2^)				
Median (IQR)	25.1 (23.3–26.8)	24.5 (22.8–26.1)	25.6 (23.8–27.6)	
Range	18.5–40.1	18.5–40.1	18.8–40.1	
CCI (value)				<0.001
Median (IQR)	0.0 (0.0)	0.0 (0.0)	0.0 (0.0)	
Mean (SD)	0.1 (0.4)	0.08 (0.4)	0.2 (0.5)	
Range	0–3	0–3	0–3	
CCI ≥ 1 [No. (%)]	58 (7.9)	13 (3.3)	45 (13.3)	<0.001
Partner’s age (years)				0.1
Median (IQR)	34.0 (32–38)	34.0 (31–38)	35.0 (32–38)	
Range	20.0–53.0	20.0–48.0	20.0–53.0	
Duration of infertility (months)				0.01
Median (IQR)	20.0 (12–30)	20.0 (12–30)	24.0 (12–36)	
Range	12.0–144.0	12.0–144.0	12.0–120.0	
Testis volume (Prader estimation)				0.7
Median (IQR)	15.0 (11–20)	15.0 (12–18)	15.0 (11–20)	
Range	2–25	2–25	3–25	
Varicocele [No. (%)]	287 (39.5)	160 (41.3)	127 (37.4)	0.4
Current smoking status [No. (%)]	426 (58.7)	223 (57.6)	203 (59.9)	0.7

Keys: TyG index = Triglycerides/glucose index; BMI = body mass index; CCI = Charlson Comorbidity Index; * *p* value according to the Mann-Whitney test for continuous data and the Chi Square Test for categorical variables, as indicated.

**Table 2:** Hormonal and metabolic of the study cohort according to TyG index suggesting insulin resistance (*N* = 726).

	Overall (*N* = 726)	TyG Index < 8.1 (*N* = 387)	TyG Index ≥ 8.1 (*N* = 339)	*p* Value *
Fasting Glucose (mg/dL)				<0.001
Median (IQR)	88.0 (83–95)	86.0 (80–91)	90.0 (85–97)	
Range	65.0–310.0	65.0–149.0	65.0–310.0	
Total cholesterol (mg/dL)				<0.001
Median (IQR)	192.0 (169–219)	182.0 (162–200)	203.0 (180–226)	
Range	112.0–362.0	112.0–284.0	113.0–362.0	
Triglycerides (mg/dL)				<0.001
Median (IQR)	86.0 (66–128)	60.0 (50–68)	115.0 (92–163)	
Range	24.0–927.0	24.0–131.0	58.0–927.4	
Insulin (mUI/L)				<0.001
Median (IQR)	7.9 (5.6–10.8)	6.6 (5.0–9.4)	9.0 (6.6–12.0)	
Range	1.3–244.1	2.0–59.0	1.3–244.1	
HOMA index				<0.001
Median (IQR)	1.7 (1.1–2.3)	1.4 (1.0–1.9)	2.0 (1.4–2.7)	
Range	0.1–75.9	0.1–14.8	0.5–75.9	
HOMA > 2.6 [No. (%)]	121 (16.6)	26 (6.7)	95 (28.0)	<0.001
FSH (mUI/mL)				0.3
Median (IQR)	6.0 (3.6–11.5)	5.7 (3.6–11.0)	6.3 (3.7–12.1)	
Range	0.1–76.3	1.0–65.6	0.1–76.3	
LH (mUI/mL)				0.8
Median (IQR)	4.4 (3.2–6.3)	4.4 (3.2–6.3)	4.4 (3.2–6.3)	
Range	0.1–77.0	0.8–36.3	0.1–77.0	
tT (ng/mL)				0.01
Median (IQR)	4.3 (3.3–5.6)	4.5 (3.4–5.7)	4.2 (3.1–5.5)	
Range	0.9–17.0	0.9–10.7	1.2–17.0	
tT < 3 ng/mL [No. (%)]	133 (17.8)	55 (14.2)	78 (23.1)	<0.01
SHBG (nmol/L)				0.01
Median (IQR)	35.0 (26–44)	38.1 (29–48)	32.0 (25–41)	
Range	11.0–170.0	11.0–154.0	12.0–170.0	
InhB (pg/mL)				0.5
Median (IQR)	114.0 (50–170)	111.2 (41–178)	115.0 (61.6–167.3)	
Range	0.5–538.0	0.5–328.0	0.5–538.0	
Prolactin (ng/mL)				0.9
Median (IQR)	8.8 (6.7–12.9)	8.8 (6.6–13.2)	8.8 (6.9–12.9)	
Range	1.0–18.7	1.0–18.7	1.0–17.7	

Keys: TyG index = Triglycerides/glucose index; HOMA = Homeostatic Model Assessment; tT = Total testosterone;.* *p* value according to the Mann-Whitney test for continuous data and the Chi-Square Test for categorical variables, as indicated.

**Table 3:** Seminal parameters of the study cohort according to TyG index suggesting insulin resistance (*N* = 726).

	Overall (*N* = 726)	TyG Index < 8.1 (*N* = 387)	TyG Index ≥ 8.1 (*N* = 339)	*p* Value *
Idiopathic NOA [No. (%)]	147 (19.7)	63 (16.3)	84 (24.8)	<0.001
Semen volume (mL)				0.8
Median (IQR)	3.0 (2–4)	3.0 (2–5)	3.0 (2–4)	
Range	0.9–13.0	0.9–10.0	1.0–13.0	
Sperm concentration				0.03
Median (IQR)	10.0 (2–29)	12.0 (2.8–30.0)	8.0 (1.5–25.0)	
Range	0.5–455.3	0.5–305.9	0.5–455.3	
Sperm concentration < 15 × 10^6^/mL [No. (%)]	426 (58.6)	205 (52.9)	221 (65.1)	0.01
Progressive motility				0.4
Median (IQR)	21.0 (10–35)	21.0 (10–35)	20.0 (9–35)	
Range	0.0–78.0	0.0–75.0	0.0–78.0	
Progressive motility < 32% [No. (%)]	507 (69.8)	264 (68.2)	243 (71.6)	0.3
Normal morphology				0.2
Median (IQR)	2.0 (1–7)	2.0 (1–6)	2.0 (1–7)	
Range	0.0–100	0.0–100.0	0.0–92.0	
Normal morphology < 4% [No. (%)]	459 (63.2)	240 (62.0)	219 (64.6)	0.5
Number of sperm alterations [No. (%)]				0.2
0	66 (9.1)	39 (10.1)	27 (7.9)	
1	188 (25.8)	109 (28.1)	79 (23.3)	
2	225 (30.9)	115 (29.7)	110 (32.4)	
3	247 (34.0)	124 (32.1)	123 (36.4)	
Sperm DNA fragmentation (SDF) index				0.01
Median (IQR)	34.5 (21.2–51.7)	28.0 (18.5–48.5)	38.2 (23.1–53.7)	
Range	0.4–99.8	0.4–97.7	0.4–99.8	
SDF index > 30% [No. (%)]	403 (55.5)	188 (48.7)	215 (63.4)	<0.01

Keys: TyG index = Triglycerides/glucose index; NOA = Non obstructive azoospermia; SDF = Sperm DNA fragmenta-tion index. * *p* value according to the Mann-Whitney test for continuous data and the Chi Square Test for categorical variables, as indicated.

**Table 4:** Logistic regression models predicting oligozoospermia, idiopathic NOA and DFI > 30% (OR; *p* value [95% CI]) in the whole cohort.

	Oligozoospermia		Idiopathic NOA		SDF > 30%	
	UVA Model	MVA Model	UVA Model	MVA Model	UVA Model	MVA Model
Age	1.15; 0.1 [0.78–1.86]	1.13; 0.7 [0.64–1.93]	1.03; 0.4 [0.73–1.11]	1.01; 0.2 [0.76–1.32]	1.11; <0.001 [1.03–1.12]	1.10; 0.01 [1.01–1.12]
BMI	1.02; 0.3 [0.97–1.07]	1.06; 0.1 [0.99–1.14]	1.10; 0.2 [0.94–1.23]	1.11; 0.3 [0.95–1.35]	0.98; 0.9 [0.89–1.05]	0.99; 0.9 [0.92–1.07]
CCI	1.18; 0.6 [0.61–2.31]	1.49; 0.4 [0.57–3.57]	2.09; <0.001 [1.55–2.82]	2.05; 0.03 [1.06–3.96]	1.09; 0.1 [0.83–1.21]	1.01; 0.1 [0.76–1.13]
Testicular volume	0.84; <0.01 [0.81–0.88]	0.89; <0.01 [0.84–0.93]	0.83; <0.001 [0.81–0.86]	0.91; <0.001 [0.86–0.96]	0.95; <0.01 [0.91–0.98]	0.93; <0.01 [0.88–0.98]
FSH	1.29; <0.001 [1.21–1.38]	1.21; <0.001 [1.13–1.30]	1.13; <0.001 [1.12–1.15]	1.11; <0.001 [1.06–1.12]	1.24; 0.1 [0.97–1.05]	1.23; 0.1 [0.76–1.58]
TyG index ≥ 8.1	1.69; <0.01 [1.18–2.41]	1.58; 0.03 [1.1–2.49]	1.69; <0.01 [1.15–2.47]	1.78; <0.01 [1.12–2.85]	1.82; <0.01 [1.21–2.74]	1.91; <0.01 [1.21–3.04]

Keys: UVA = Univariate model; MVA = Multivariate model, TyG index = Triglycerides/glucose index; BMI = body mass index; CCI = Charlson Comorbidity Index; NOA = non-obstructive azoospermia; SDF = Sperm DNA Fragmentation index.

## Data Availability

The data that support the findings of this study are available on request from the corresponding author, A.S. The data are not publicly available due to their containing information that could compromise the privacy of research participants.
